# Bisphenol A-responsive microgel comprising hydrophilic poly(acrylamide) network

**DOI:** 10.1080/14686996.2025.2610881

**Published:** 2026-01-12

**Authors:** Akifumi Kawamura, Fumiya Tanaka, Yuriko Nishimura, Takashi Miyata

**Affiliations:** a Department of Chemistry and Materials Engineering, Kansai University, Suita, Osaka, Japan; b Organization for Research and Development of Innovative Science and Technology, Kansai University, Suita, Osaka, Japan

**Keywords:** Molecule-responsive microgel, inverse miniemulsion polymerization, water-soluble emulsifier, RAFT polymerization, zwitterionic polymer, inclusion complex, cyclodextrin

## Abstract

Stimuli-responsive microgels that exhibit rapid changes in size in response to external stimuli such as pH and temperature are of great interest for the development of smart sensors, drug delivery carriers, and separation materials. This paper describes the preparation of molecule-responsive microgels with molecular recognition sites comprising a hydrophilic network *via* inverse miniemulsion polymerization using a water-soluble emulsifier. The water-soluble emulsifier comprising hydrophilic poly(sulfobetaine) and hydrophilic/oleophilic poly[oligo(ethylene glycol)methacrylate-*co*-2-(2’−methoxyethoxy)ethyl methacrylate] blocks were synthesized *via* reversible addition fragmentation chain transfer (RAFT) polymerization. The resulting block copolymer, PSB-POEG, stabilized the water-chloroform interface in a water-in-oil (W/O) emulsion. Water droplets in a W/O emulsion stabilized with PSB-POEG allowed the inverse miniemulsion polymerization of acrylamide (AAm), acryloyl-modified β-cyclodextrin (CD), and *N,N’*-methylenebisacrylamide to obtain CD-conjugated PAAm microgels with a diameter of approximately 150 nm. The resulting CD-PAAm microgels were stably dispersed in an aqueous medium by the addition of water, followed by evaporation of chloroform. The CD-PAAm microgels exhibited rapid shrinkage in response to bisphenol A (BPA) owing to the formation of CD-BPA-CD complexes acting as dynamic cross-links. The proposed method for preparing hydrophilic microgels by inverse miniemulsion polymerization using a water-soluble emulsifier allows the preparation of molecularly imprinted and bioconjugated microgels, providing a useful platform for designing rapidly responsive soft nanomaterials for molecular sensors, separation substrates, and drug delivery carriers.

## IMPACT STATEMENT

Microgels comprising polyacrylamide networks with β-cyclodextrin ligands were synthesized via inverse miniemulsion polymerization using water-soluble emulsifiers. Resulting microgels were easily dispersed in water and shrank upon the addition of BPA.

## GRAPHICAL ABSTRACT

**Figure:**
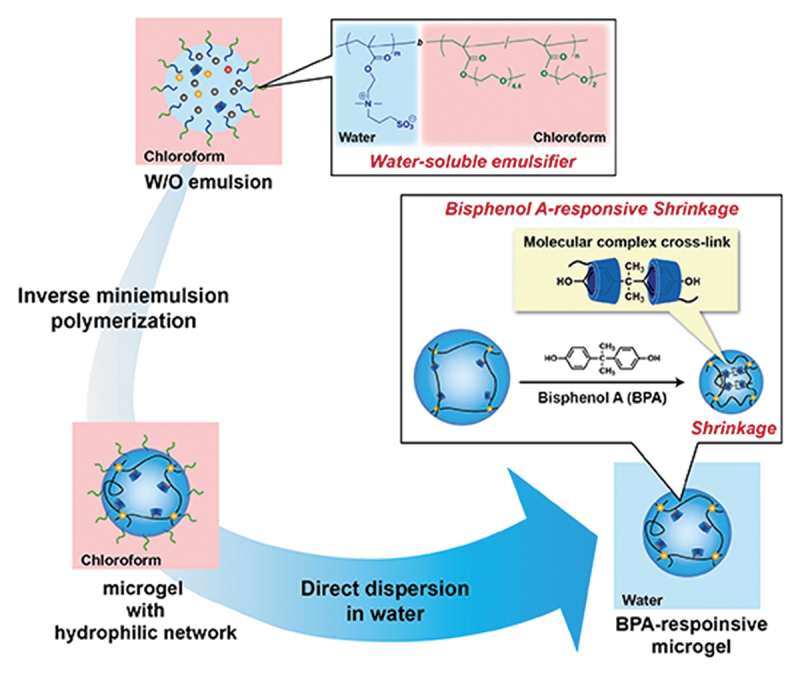

## Introduction

Stimuli-responsive microgels that undergo changes in size in response to external stimuli are of great interest because of their potential applications in drug delivery systems, smart separation substrates, and sensors [1–4]. The stimuli-responsiveness of such microgels is mainly based on changes in the hydrophilicity of the polymer networks or osmotic pressure by charged groups. In addition, bioconjugation onto stimuli-responsive microgels allows the formation of biomolecule-responsive microgels [5–9]. For example, glucose oxidase-immobilized poly(acrylic acid) microgels showed a glucose-triggered decrease in size owing to the protonation of carboxylates of microgels by a decrease in local pH, resulting from the enzymatic reaction of glucose oxidase [8].

In contrast to conventional stimuli-responsive hydrogels, we have developed molecularly stimuli-responsive hydrogels [10–13] and microgels [14–18] that change in size upon recognition of a target molecule. The recognition of a target molecule induces a change in the crosslinking density through the formation or dissociation of molecular complex cross-links. For example, a polyacrylamide (PAAm) hydrogel with β-cyclodextrin (CD) as a ligand shrank in response to the target bisphenol A (BPA) owing to the formation of sandwich-like CD-BPA-CD complexes acting as dynamic cross-links [13]. Moreover, molecular imprinting of BPA during the preparation of BPA-responsive hydrogels allows for greater shrinkage than that of non-imprinted hydrogels. We also revealed that a rapid responsive change in size was accomplished by decreasing the size of the molecule-responsive hydrogel [14–18]. Because surfactants that stabilize the interface of monomer droplets and the resulting microgels may affect the molecular complexes as dynamic cross-links, soap-free emulsion polymerization was applied to prepare molecule-responsive microgels. The glucose-responsive microgel prepared *via* soap-free emulsion copolymerization of diethylaminoethyl methacrylate, methacrylate with a pendant glucose, acryloyl-conjugated lectin concanavalin A, and poly(ethylene glycol)dimethacrylate exhibited the target glucose-responsive swelling [15].

Microgels prepared *via* soap-free emulsion polymerization are composed of a relatively hydrophobic polymer network. Such hydrophobic polymer networks induce nonspecific adsorption of hydrophobic molecules onto microgels through hydrophobic interactions, resulting in undesirable shrinkage. Therefore, molecule-responsive microgels with hydrophilic polymer networks are required to recognize a hydrophobic target molecule without nonspecific adsorption. Inverse miniemulsion polymerization is a promising method for preparing microgels with hydrophilic networks [19]. For example, PAAm [20–24], poly[oligo(ethylene glycol)methacrylate] (POEGMA) [25,26], and zwitterionic polycarboxybetaine microgels [27] were prepared by inverse miniemulsion polymerization. As amphiphilic surfactants were used to stabilize the aqueous monomer solution droplets in the W/O emulsion, the surfaces of the resulting hydrophilic microgels were covered with surfactants. The dispersion of the resulting microgels in an aqueous medium requires the removal of adsorbed surfactants from the surface of the resulting microgels by thorough washing with polar solvents such as methanol and tetrahydrofuran. Therefore, there is a demand for a method for the facile preparation of water-dispersible microgels composed of a hydrophilic polymer network without removal of the surfactant by a thorough washing process.

Recently, we proposed a novel strategy for preparing nanocapsules [28,29] and core-shell microgels [30] comprising a completely hydrophilic polymer network *via* interfacial reaction in a W/O emulsion. We designed a water-soluble emulsifier composed of extremely hydrophilic poly(2-methacryloyloxyethyl phosphorylcholine) (PMPC) and hydrophilic/oleophilic POEGMA blocks. The water-soluble emulsifier PMPC-*b*-POEGMA stabilized the water-chloroform interface because the PMPC and POEGMA blocks were distributed in the water and chloroform phases, respectively. Water droplets stabilized with a PMPC-*b*-POEGMA emulsifier in a W/O emulsion provide a useful interfacial reaction field for preparing soft nanomaterials. The copolymerization of poly(ethylene glycol)methacrylate with bis(2-methacryloyl)oxyethyl disulfide from the water-soluble PMPC-*b*-POEGMA emulsifier, which stabilized the water-chloroform interface in the W/O emulsion, provided reductively responsive nanocapsules [28]. Nanocapsules were also prepared by the interfacial cross-linking of a PMPC-*b*-POEGMA emulsifier using divinyl sulfone [29]. Moreover, the copolymerization of MPC with *N,N’*-methylenebisacrylamide (MBAA) from the water-soluble PMPC-*b*-POEGMA emulsifier in water droplets of the W/O emulsion allowed the preparation of a core-shell microgel with a hydrophilic PMPC core and LCST-type thermoresponsive POEGMA shell derived from the PMPC-*b*-POEGMA emulsifier [30]. The most fascinating advantage of the preparation method of soft nanomaterials using a W/O emulsion stabilized with a water-soluble PMPC-*b*-POEGMA emulsifier is that the resulting soft nanomaterials are easily dispersed in aqueous media without the need for thorough washing to remove the emulsifier. Therefore, this method is promising for preparing molecule-responsive microgels composed of hydrophilic networks with molecular complexes as dynamic cross-links.

To address the challenges associated with nonspecific adsorption and complex surfactant removal in previous methods, this paper reports a novel method for preparing BPA-responsive microgels comprising a hydrophilic PAAm network *via* inverse miniemulsion polymerization using a water-soluble emulsifier. A water-soluble block copolymer comprising a hydrophilic poly(sulfobetaine) block and a hydrophilic/oleophilic poly[oligo(ethylene glycol)methacrylate-*co*-2-(2’−methoxyethoxy)ethyl methacrylate] (PSB-POEG) was synthesized *via* reversible addition fragmentation chain transfer (RAFT) polymerization, and its interfacial properties were investigated to prepare W/O emulsions. BPA-responsive microgels were prepared *via* inverse miniemulsion polymerization of AAm, acryloyl-CD, and MBAA using PSB-POEG as a water-soluble emulsifier. This study also describes the BPA-responsive behavior of the resulting BPA-responsive microgels. Fundamental research on the preparation of microgels with molecular recognition sites comprising a hydrophilic network will contribute significantly to the development of molecular sensors, separation materials, and carriers for drug delivery systems.

## Materials and methods

### Materials

Acrylamide (AAm), *N,N*-diethylaminoethyl methacrylate, *N,N’*-methylenebisacrylamide (MBAA), 2,2’-azobis[2-(2-imidazolin-2-yl)propane]dihydrochloride (VA-044), 4,4’-azobis(4-cyanovaleric acid) (ACVA), bisphenol A (BPA), *p*-toluenesulfonyl chloride, *p*-toluenesulfonic acid monohydrate, β-cyclodextrin (CD), sodium azide, triphenyl phosphine, and acryloyl chloride were purchased from Fujifilm Wako Pure Chemical Corporation (Osaka, Japan). 1,3-propanesultone, 2-(2’−methoxyethoxy)ethyl methacrylate (MEO_2_MA), poly(ethylene glycol)dimethacrylate, and 2,2,2-trifluoroethanol (TFE) were purchased from Tokyo Chemical Industry Co., Ltd. (Tokyo, Japan). Oligo(ethylene glycol)methacrylate (*M*_w_: 300; OEGMA), and 4-cyano-4-(phenylcarbonothioylthio)pentanoic acid (CTPA) were purchased from Millipore-Sigma (St. Louis, MO, U.S.A.). [2-(Methacryloyloxy)ethyl]dimethyl-(3-sulfopropyl)ammonium hydroxide (SB) [31] and monoacryloyl-β-cyclodextrin (acryloyl-CD) [13] were prepared as previously reported. The detailed synthesis is described in the Supporting Information. OEGMA and MEO_2_MA were purified using a basic alumina column to remove inhibitors. All the aqueous solutions were prepared using ultrapure water (Milli-Q, 18.2 MΩ·cm). Other solvents were obtained from commercial sources and used as received.

### Synthesis of PSB macroRAFT agent

SB (1.00 g, 3.58 mmol), CTPA (20.4 mg, 0.073 mmol), and ACVA (4.0 mg, 0.014 mmol) were dissolved in 2.5 mL TFE. The solution was deoxygenated by three freeze – pump – thaw cycles, followed by stirring at 90°C for 24 h. The product was precipitated in methanol (50 mL) to remove the unreacted monomers. The isolated product was dried under vacuum to obtain PSB macroRAFT agent (yield: 0.78 g).

### Synthesis of PSB-b-P(OEGMA-co-MEO_2_MA) (PSB-POEG)

OEGMA (370 mg, 1.24 mmol), MEO_2_MA (237 mg, 1.26 mmol), PSB macroRAFT agent (200 mg, 12.6 μmol), and ACVA (706 μg, 2.52 μmol) were dissolved in 3.9 mL of TFE. The solution was deoxygenated by bubbling with Ar gas for 30 min and stirred at 90°C for 8 h. The product was precipitated in a 78 mL diethyl ether/hexane mixture (diethyl ether/hexane = 2/1 (v/v)) to remove the unreacted monomers. The isolated polymer product was dried under vacuum to obtain PSB-*b*-P(OEGMA-*co*-MEO_2_MA) (PSB-POEG; 0.49 g, 61% yield).

### Preparation of W/O emulsion using PSB-POEG as an emulsifier

Chloroform (9.9 mL) was added to 0.1 mL of phosphate buffered saline (PBS(–)) containing 15 mg of PSB-POEG, followed by sonication using an ultrasonic homogenizer (Sonifier SFX250, Branson Ultrasonics Corp., CT, U.S.A.) at 20% amplitude for 3 min.

### Preparation of PAAm and CD-PAAm microgels by inverse miniemulsion polymerization

Acryloyl-CD, AAm, MBAA, VA-044, and PSB-POEG (15 mg) were dissolved in 0.1 mL PBS (–). To 9.9 mL of chloroform, a PBS(–) solution containing all components of the inverse miniemulsion polymerization was added, and the water-chloroform mixture was sonicated using an ultrasonic homogenizer (Sonifier SFX250, Branson Ultrasonics Corp., CT, U.S.A.) at 20% amplitude for 3 min to form a W/O emulsion. The polymerization was performed at 45°C for 5 h. After adding 10 mL of water to the reaction mixture, the chloroform was removed under reduced pressure. The resulting aqueous dispersion of microgels was poured into seamless cellulose tubing (Biotech CE Dialysis Tubing, molecular cut-off: 1000 kDa, Repligen Corp., MA, U.S.A.) and dialyzed against ultrapure water. The PAAm microgel was synthesized using a method similar to that of the CD-PAAm microgel, without acryloyl-CD. The synthesis conditions are summarized in Table 1.

**Table 1.** Synthesis condition of CD-PAAm microgels.

CD content (%)	AAm	acryloyl-CD	MBAA	VA-044
0	4.5 mg (63 μmol)	–	16 μg (0.10 μmol)	36 μg (0.11 μmol)
5.0	4.2 mg (60 μmol)	3.6 mg (3.0 μmol)	16 μg (0.10 μmol)	36 μg (0.11 μmol)
9.8	4.0 mg (57 μmol)	7.4 mg (6.2 μmol)	16 μg (0.10 μmol)	36 μg (0.11 μmol)

### Characterization

#### GPC measurements

GPC measurements were conducted using a Shimadzu Prominence HPLC system (Shimadzu Corp., Kyoto, Japan) equipped with PFG 100 Å and PFG 1000 Å columns (Polymer Standards Service GmbH, Germany) at 40°C under a flow rate of 1.0 mL•min^−1^ using a refractive index detector. TFE solution containing 20 mM sodium trifluoroacetate was used as the eluent. *M*_n_, *M*_w_, *and M*_w_/*M*_n_ (*Ð*) were calculated using near-monodisperse poly(methyl methacrylate) standards.

#### Dynamic light scattering measurements

Dynamic light scattering (DLS) measurements were conducted at 25°C using an ELS-Z1000 spectrometer (Otsuka Electronics Co., Ltd., Osaka, Japan) equipped with a He – Ne laser (λ = 633.8 nm). The detection angle was fixed at 165°. The diameter and polydispersity index were calculated using the cumulant method, and the size distribution was obtained using Marquardt analysis.

#### Interfacial tension measurements

Interfacial tension was measured using the pendant drop technique using a contact angle meter (DMo-502, Kyowa Interface Science Co., Ltd., Saitama, Japan). A pendant drop of PBS(–) containing PSB-POEG was prepared in chloroform at 25°C using an 18 G teflon-coated needle. The interfacial tension was determined using the Young-Laplace Equation (1).

(1) $$\Delta \rho =\gamma \cdot \left(1/r_{1}+1/r_{2}\right)$$

where Δρ is the density difference, *γ* is the interfacial tension, and r_1_ and r_2_ are the radii of curvature of the surface [32].

#### Swelling measurements

To a 2.0 mL aqueous CD-PAAm microgel dispersion, 1.0 mL of an aqueous BPA solution (120 mg/L) was added. The diameters of the CD-PAAm microgels were measured using DLS. The swelling ratio, (*V*/*V*_0_) of the CD-PAAm microgels was determined using Equation (2)

(2) $$\mathrm{swelling}\;\mathrm{ratio}=V/V_{0}=\left(d/d_{0}\right)3$$

where *d* is the cumulant diameter of the CD-PAAm microgels in the presence of BPA at various incubation time, and *d*_0_ is the cumulant diameter of the CD-PAAm microgels in the absence of BPA.

## Results and discussion

### Synthesis of PSB-POEG

**Figure 1.**
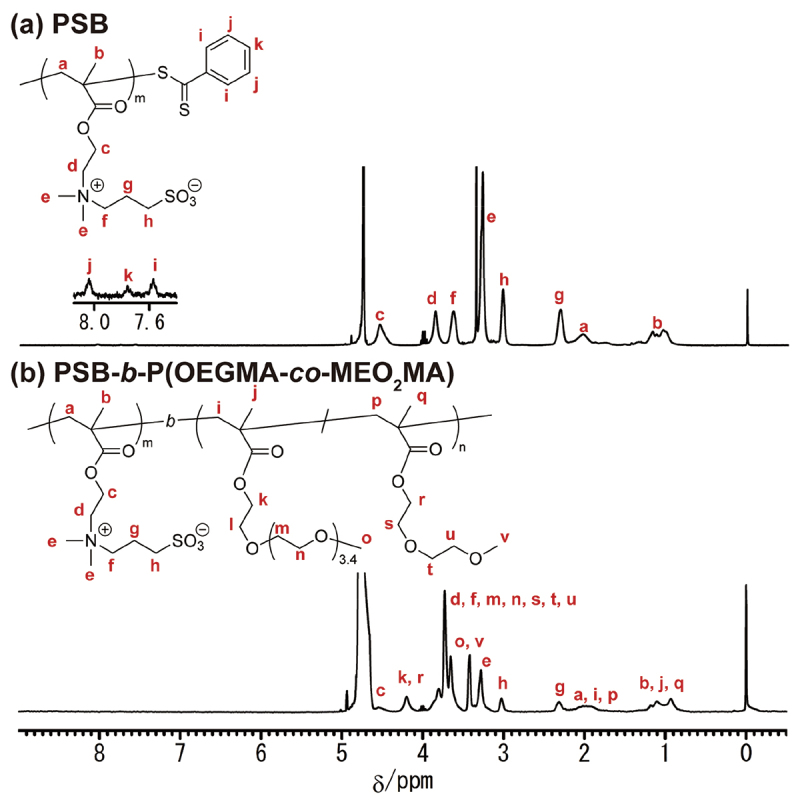
^1^H NMR spectra of (a) PSB and (b) PSB-POEG. (400 MHz, D_2_O).

**Figure 2.**
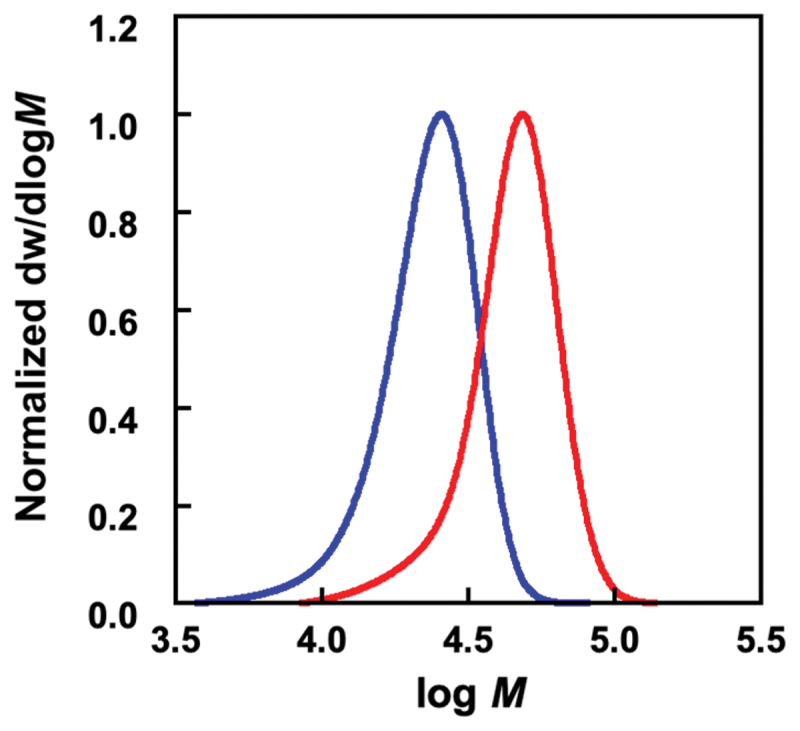
Molecular weight distributions of PSB (blue) and PSB-POEG (red).

**Scheme 1.**
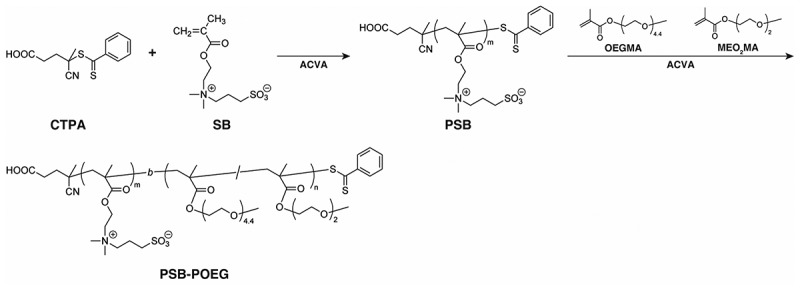
Synthesis of PSB-POEG.

In conventional inverse miniemulsion polymerization, an amphiphilic emulsifier is used to stabilize the water-oil interface. To ensure stable dispersion in aqueous media, emulsifiers adsorbed on the surface of the resulting microgels are removed by thorough washing with a polar solvent. Our water-soluble emulsifier, consisting of an extremely hydrophilic zwitterionic polymer block and a hydrophilic/oleophilic polymer block with an oligo(ethylene glycol) side chain, facilitates the stabilization of water-chloroform interfaces in W/O emulsions and its removal from the surface of the resulting microgels through simple dialysis. In this study, we designed a water-soluble block copolymer comprising a hydrophilic PSB and a hydrophilic/oleophilic P(OEGMA-*co*-MEO_2_MA) block as an emulsifier for inverse miniemulsion polymerization. In contrast to our previous water-soluble emulsifier, PMPC-*b*-POEGMA, PSB was employed as the hydrophilic block of a water-soluble emulsifier instead of PMPC to ensure the versatility of the zwitterionic polymer block within the water-soluble emulsifier. Copolymers of OEGMA and MEO_2_MA exhibit an LCST-type phase transition [33]. The LCST decreases with increasing MEO_2_MA composition in P(OEGMA-*co*-MEO_2_MA) because of the hydrophobic nature of MEO_2_MA. Therefore, the copolymerization of OEGMA with MEO_2_MA was expected to assist in the distribution of the P(OEGMA-*co*-MEO_2_MA) block of the water-soluble emulsifier into chloroform. In fact, we previously succeeded in the formation of W/O emulsion using PMPC-*b*-P(OEGMA-*co*-MEO_2_MA) as a water-soluble emulsifier [30]. PSB-*b*-P(OEGMA-*co*-MEO_2_MA) (PSB-POEG) was synthesized *via* the RAFT polymerization of SB, followed by that of OEGMA and MEO_2_MA (Scheme 1). Figure 1(a) shows the ^1^H NMR spectrum of PSB. By comparing the integration of the dithiobenzoyl protons of the RAFT agent terminus (8.1–7.5 ppm, protons i, j, and k) with the methylene protons next to the sulfonate group of PSB (3.0 ppm, proton h), the polymerization degree of PSB (*D*_p PSB_) was determined to be 47. Using the resulting PSB as a macroRAFT agent, PSB-POEG was synthesized *via* RAFT copolymerization of OEGMA and MEO_2_MA. Figure 1(b) shows the ^1^H NMR spectrum of PSB-POEG. Characteristic resonances for both PSB and P(OEGMA-*co*-MEO_2_MA) were observed, including signals attributed to the methyl protons of the dimethylammonium groups of PSB (3.3 ppm, proton e) and methoxy protons of P(OEGMA-*co*-MEO_2_MA) (3.4 ppm, protons o and v). The molecular weight distribution of PSB shifted toward a higher molecular weight without a shoulder derived from unreacted PSB after the RAFT copolymerization of OEGMA and MEO_2_MA using a PSB macroRAFT agent (Figure 2). By comparing the overall integration of the methylene proton of both OEGMA and MEO_2_MA (4.2 ppm, protons k and r) with the overall integration of the 3.9–3.5 ppm region (protons d, f, m, n, s, t, and u), in which 17.6 protons of OEGMA, 6 protons of MEO_2_MA, and 4 protons of SB are resonated, the *F*_OEGMA_ of P(OEGMA-*co*-MEO_2_MA) was determined to be 0.64. The polymerization degree of P(OEGMA-*co*-MEO_2_MA) (*D*_p POEG_), obtained by integrating the methylene resonance of P(OEGMA-*co*-MEO_2_MA) at 4.2 ppm (protons k and r) against the methylene resonance of PSB at 3.0 ppm (proton h), was determined to be 105. Table 2 summarizes results of the PSB and PSB-POEG synthesis.

**Table 2.** Results of the synthesis of PSB macroRAFT agent and PSB-POEG.

	*F*_OEGMA_^a^	*D*_p_^a^		*M*_n_ (×10^4^) ^a^	*M*_n_ (×10^4^) ^b^	*M*_w_ (×10^4^) ^b^	*Ð* ^b^
		PSB	P(OEGMA-*co*-MEO_2_MA)				
PSB macroRAFT agent	–	47	–	1.31	2.12	2.43	1.15
PSB-POEG	0.64	47	105	4.04	4.13	4.70	1.14

^a^calculated from ^1^H NMR; ^b^Calculated from GPC curves using near-monodisperse poly(methyl methacrylate) calibration standards.

### W/O emulsion formation using PSB-POEG as a water-soluble emulsifier

**Figure 3.**
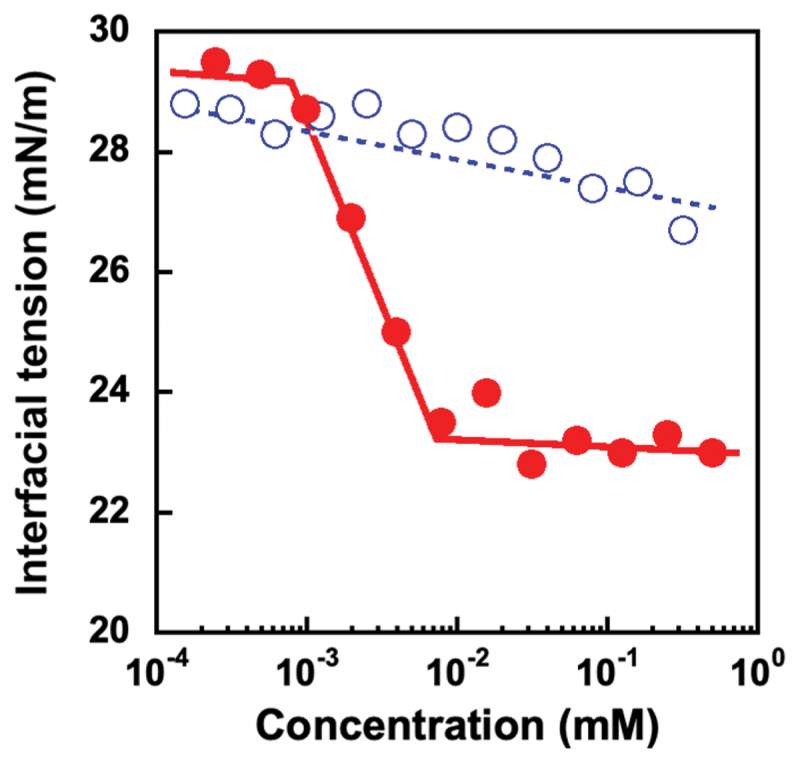
Change in the interfacial tension of water-chloroform interface as a function of the concentration of PSB 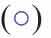 and PSB-POEG 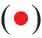.

PSB dissolves in a limited number of solvents, including water, trifluoroethanol, hexafluoroisopropanol, and ionic liquids. The solution properties of PSB are influenced by several factors, such as the concentration, molecular weight, and ionic strength [34–36]. PSB with a molecular weight greater than 20 kDa exhibits an upper critical solution temperature (UCST)-type phase transition owing to intra- and intermolecular dipole-dipole interactions. The addition of salt induces the shielding of dipole-dipole interactions, resulting in the dissolution of high molecular weight PSB in water. In this study, phosphate buffered saline (PBS (–)) with an ionic strength of 160 mM was used to dissolve and distribute the PSB block of PSB-POEG in the water phase. Figure 3 shows the effect of PSB and PSB-POEG concentrations on the interfacial tension between water and chloroform. The interfacial tension between water and chloroform slightly decreased with increasing PSB concentration. In contrast, the interfacial tension decreased drastically above 0.91 μM of PSB-POEG and remained unchanged above 7.9 μM. The drastic decrease in the interfacial tension indicates that PSB-POEG stabilizes the interface between water and chloroform by distributing the PSB and P(OEGMA-*co*-MEO_2_MA) blocks in the water and chloroform phases, respectively. The interfacial tension and critical micelle concentration (CMC) were approximately 23 mN/m and 7.9 μM, respectively. These values are comparable to those of a previously reported PMPC-*b*-P(OEGMA-*co*-MEO_2_MA) system (i.e. the interfacial tension and CMC were 20 mN/m and 5.6 μM, respectively) [30]. This result indicates that PSB-POEG acts as a surfactant in the water-chloroform two-phase system, exhibiting the comparable surface activity with PMPC-*b*-POEGMA, as previously reported by our group [28–30]. In addition, the effect of *D*_p POEG_ of PSB-POEGs on CMC was evaluated in a water-chloroform two-phase system (Figure S1). As a result, CMC decreased with increasing *D*_p POEG_ and remained unchanged at *D*_p POEG_ > 105. Therefore, PSB-POEG having *D*_p PSB_ of 48 and *D*_p POEG_ of 105 is suitable for preparing a W/O emulsion in a water-chloroform two-phase system. The interfacial activity measurements revealed that the combination of an extremely hydrophilic zwitterionic polymer and hydrophilic/oleophilic POEGMA is essential for designing a water-soluble emulsifier that stabilizes the water-chloroform interface.

**Figure 4.**
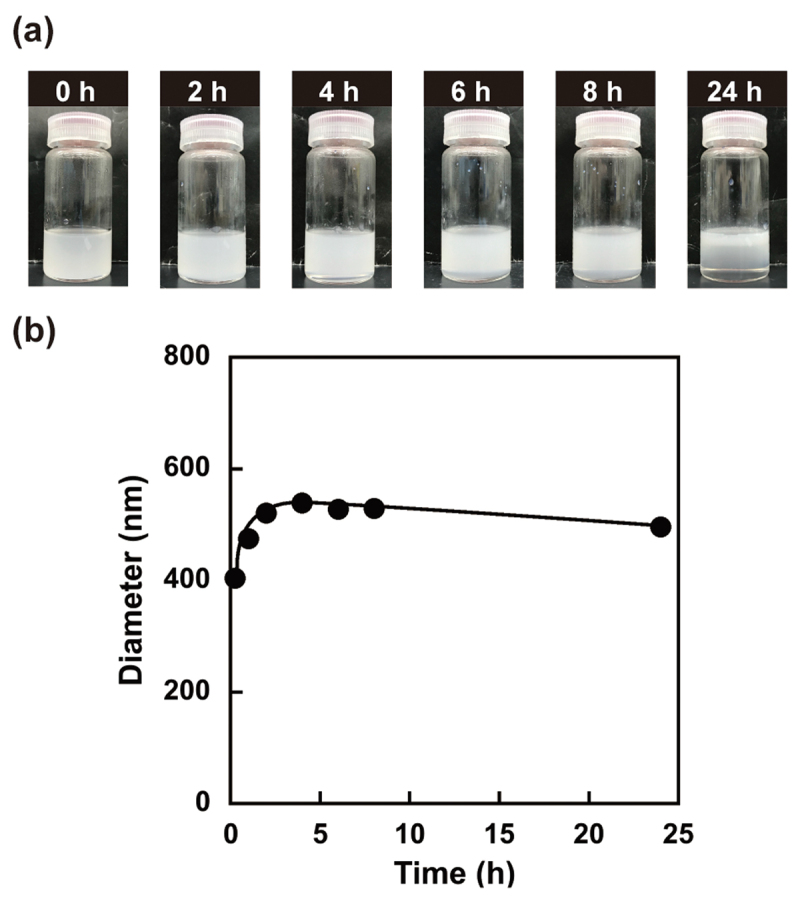
(a) Photographs of the sonicated PBS(–)/chloroform two-phase system containing the PSB-POEG emulsifier. (b) Changes in the diameter of water droplets stabilized with the PSB-POEG emulsifier in the W/O emulsion as a function of time after sonication.

The W/O emulsion was prepared by adding a PBS (–) solution containing PSB-POEG to chloroform, followed by sonication using a probe-type sonicator. Figure 4(a) shows photographs of the W/O emulsion prepared using PSB-POEG in a water-chloroform two-phase system. The milky appearance after sonication of the water-chloroform two-phase system in the presence of PSB-POEG indicates the successful formation of a W/O emulsion. On the other hand, the obvious creaming was observed after incubation for 24 h at room temperature. The size of the water droplets was evaluated using dynamic light scattering (DLS) equipped with backscatter optics. Figure 4(b) shows the change in the diameter of water droplets in the W/O emulsion prepared using PSB-POEG as a function of time after sonication. The diameter of the water droplets increased after sonication and reached equilibrium after 5 h with a diameter of approximately 500 nm. Although a slight increase in the size of the water droplets was observed in the initial stage, the water droplets in the W/O emulsion did not merge to induce phase separation after 5 h (Figure 4(a)). Despite the creaming induced by the strong buoyant force of relatively large water droplets, the PSB-POEG stabilized the water-chloroform interface and prevented the coalescence of water droplets, acting as an emulsifier. Water droplets covered with PSB-POEG emulsifiers in a W/O emulsion provide a submicron-scale reaction vessel for the preparation of smart microgels with a hydrophilic gel network.

### Preparation of PAAm microgels with CD ligands (CD-PAAm microgels)

**Figure 5.**
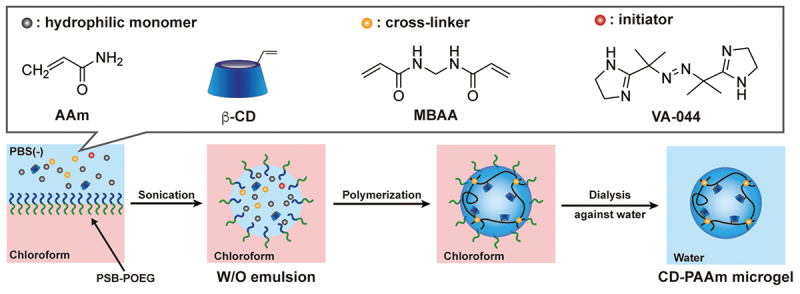
Schematic image of the preparation of the CD-PAAm microgel *via* inverse miniemulsion polymerization.

**Figure 6.**
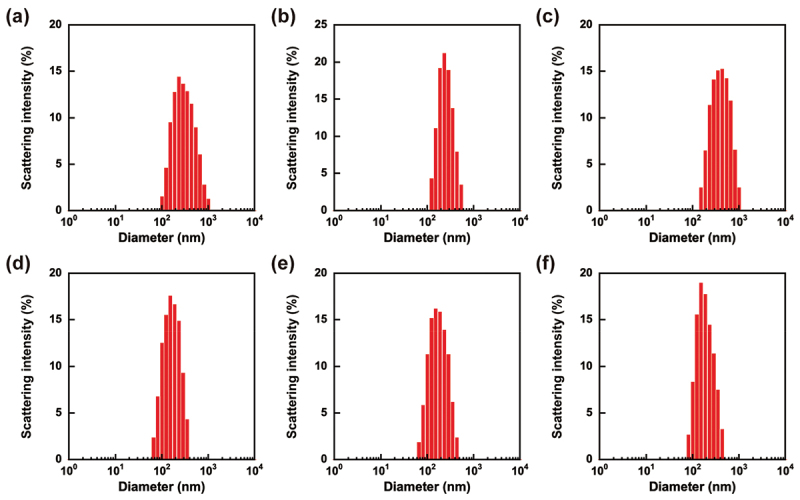
Size distributions of water droplets containing monomers and an initiator dispersed in a chloroform continuous phase (a–c) and CD-PAAm microgels in water (d–f). The CD contents were 0 (a, d), 5.0 (b, e), and 9.8 (c, f).

Water droplets stabilized with PSB-POEG as a water-soluble emulsifier in the W/O emulsion provide a submicron-scale reaction vessel that enables the polymerization of water-soluble monomers and cross-linkers dissolved in water droplets to obtain microgels with a hydrophilic polymer network. In this study, AAm, acryloyl-CD, and MBAA were copolymerized in water droplets in a W/O emulsion to obtain CD-PAAm microgels composed of a hydrophilic PAAm microgel network with CD ligands (Figure 5). In conventional inverse miniemulsion polymerization, an oleophilic radical initiator is typically employed. In this study, the water-soluble radical initiator VA-044 was applied to prevent chain elongation from the PSB-POEG emulsifiers with the RAFT agent terminus located in a chloroform continuous phase. After polymerization, the chloroform continuous phase was exchanged with water, followed by dialysis to remove the PSB-POEG emulsifier. Figure 6 shows the size distribution of the resulting CD-PAAm microgel in water. The water droplets containing monomers and an initiator had a monomodal size distribution with cumulant diameters of 230–350 nm (Figure 6(a–c), Table 3). The diameter of the water droplets containing monomers and an initiator in the W/O emulsion was smaller than that of the water droplets in the W/O emulsion prepared using PBS (–) (i.e. approximately 500 nm). The decrease in the size of the water droplets implied that the monomers and initiator dissolved in the water droplets acted as lipophobes to support emulsification. After polymerization of monomers in water droplets, the chloroform continuous phase was exchanged with water. Figure 6(d–f) show the size distributions of the CD-PAAm microgels with CD contents of 0 (i.e. PAAm microgel), 5.0, and 9.8 mol%. The monomodal size distributions of the resulting CD-PAAm microgels indicate that PAAm, acryloyl-CD, and MBAA were copolymerized in the individual water droplets of the W/O emulsion without undesirable interparticle cross-linking and fusion of water droplets during polymerization. The cumulant diameter of the CD-PAAm microgels was smaller than that of the water droplets before polymerization (Table 3). In general, polymerization in an appropriately formulated miniemulsion with a high monomer concentration yields polymer particles that are similar in size to the initial droplets [37]. However, our lower monomer concentration (2–3 times less than conventional inverse miniemulsion polymerization) was insufficient to grow the microgel network within the entirety of the initial droplets [38,39]. These results indicate that water-dispersible submicron-scale CD-PAAm microgels were successfully prepared by inverse miniemulsion polymerization using a water-soluble PSB-POEG emulsifier.

**Table 3.** Cumulant diameter and polydispersity index of monomer solution droplets in W/O emulsion and CD-PAAm microgels.

CD content (%)	Monomer solution droplets in chloroform (Before polymerization)		Microgels in water (After polymerization)	
	Diameter (nm)	Polydispersity index	Diameter (nm)	Polydispersity index
0	261	0.207	146	0.209
5.0	230	0.112	151	0.183
9.8	346	0.186	169	0.170

### BPA-responsive behavior of CD-PAAm microgels

**Figure 7.**
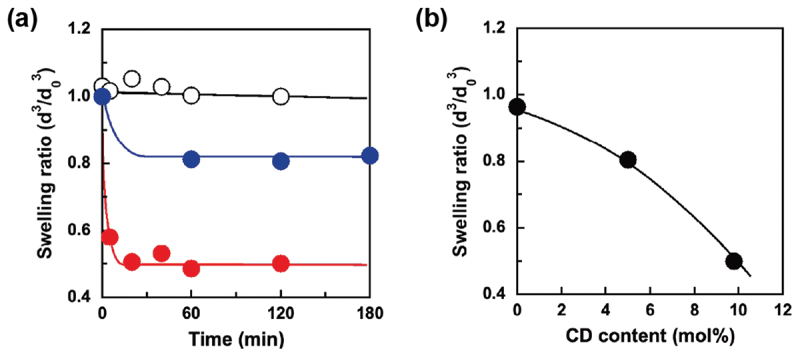
(a) Changes in the swelling ratios of CD-PAAm microgels with CD contents of 0 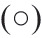, 5.0 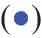 and 9.8 mol% 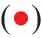 as a function of time after the addition of BPA. The concentration of BPA in the microgel dispersion was 40 mg/L. (b) Effect of CD content on the equilibrium swelling ratio of PAAm and CD-PAAm microgels in the presence of BPA (40 mg/L).

**Figure 8.**
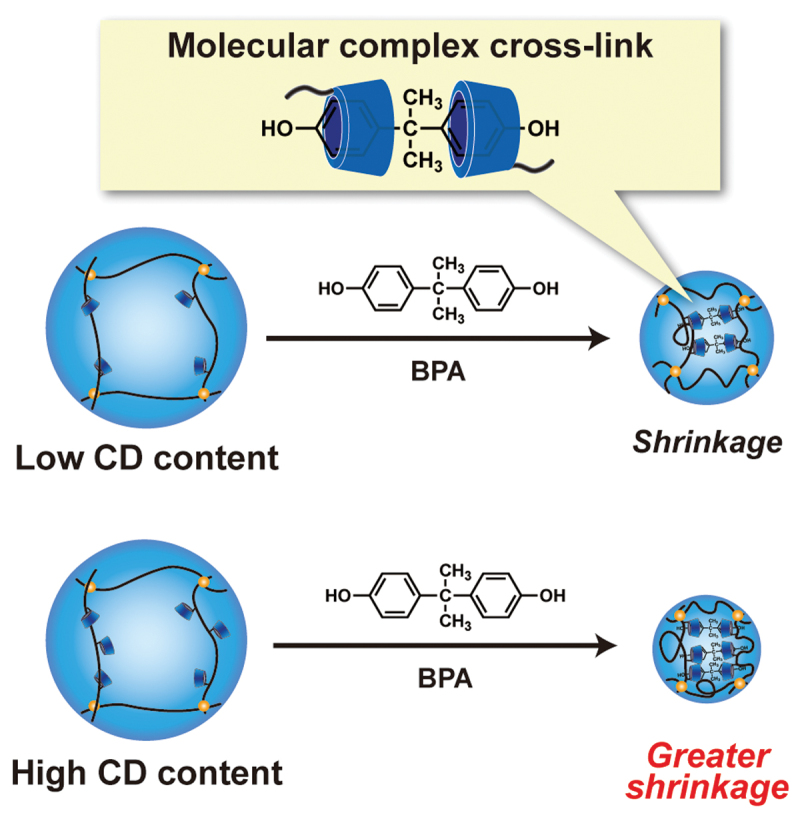
Schematic illustration of the BPA-responsive behavior of CD-PAAm microgels.

BPA is an important industrial chemical that is used as a monomer in the manufacture of polycarbonate plastics and epoxy resins. We prepared BPA-responsive soft materials containing CD ligands, such as BPA-responsive hydrogels [13], microgel valves [16], and organic-inorganic hybrid nanoparticles [17]. These BPA-responsive soft materials change in size in response to BPA because the CDs form sandwich-like CD-BPA-CD complexes with BPA, resulting in an increase in the cross-linking density. The macroscale BPA-responsive hydrogel, as previously reported by our group, required approximately 20 h to reach equilibrium swelling after the addition of BPA. In general, the swelling and shrinkage of hydrogels are governed by diffusion-limited transport of polymer chains in water. Therefore, a decrease in the size of the stimuli-responsive hydrogel allows rapid changes in its size owing to an increase in the surface area. Therefore, rapid BPA-responsiveness can be accomplished using CD-PAAm microgels with a size of approximately 150 nm prepared *via* inverse miniemulsion polymerization using a water-soluble PSB-POEG emulsifier. Figure 7(a) shows the changes in the swelling ratio of CD-PAAm microgels with CD contents of 0, 5, and 9.8 mol% as a function of time after the addition of BPA. Here, a swelling ratio of less than 1.0 means that the microgel shrinks in response to BPA. The microgel without the CD ligands remained unchanged in the presence of BPA. In contrast, the CD-PAAm microgels shrank immediately after the addition of BPA and attained equilibrium swelling within 20 min. The equilibrium swelling ratio of CD-PAAm microgels in the presence of BPA decreased with increasing CD content (Figure 7(b)). We previously reported that a macroscale PAAm hydrogel without CD ligands adsorbed only a minute amount of BPA, resulting in no change in size upon exposure to BPA [13]. Therefore, the unchanged swelling ratio of the microgel without CD ligands (i.e. PAAm microgel) upon exposure to BPA indicates a minute amount of nonspecific adsorption of BPA. We examined the effect of BPA addition on the size of poly(diethylaminoethyl methacrylate) (PDEA) microgels with and without CD ligands prepared *via* soap-free emulsion polymerization (Figure S2). Regardless of the presence of CD ligands, the resulting PDEA microgels shrank in the presence of BPA owing to the nonspecific adsorption of BPA onto the PDEA microgel network through hydrophobic interactions (Figure S3). These results indicate that the prevention of nonspecific adsorption by hydrophilic gel network is essential for recognizing a hydrophobic target molecule. The shrinkage behavior of the CD-PAAm microgels prepared in this study can be explained by the tentative model schematically illustrated in Figure 8. When BPA was added to the CD-PAAm microgel dispersion, it diffused easily into the CD-PAAm microgel. The CD ligands in the CD-PAAm microgels formed sandwich-like CD-BPA-CD complexes with BPA, which acted as dynamic cross-links. Consequently, the cross-linking density of the CD-PAAm microgels increased, resulting in shrinkage. Moreover, the increase in the CD content of the CD-PAAm microgels resulted in an increase in the cross-linking density based on complex formation between CD and BPA. Therefore, the CD-PAAm microgel with a CD content of 9.8 mol% exhibited greater shrinkage than that with a CD content of 5.0 mol% in response to BPA. Our previous study on the BPA-responsive macroscale hydrogel revealed that the elastic modulus increased after immersion in an aqueous BPA solution owing to the increase in the cross-linking density based on the CD-BPA-CD complex formation [13]. Although the changes in elasticity of CD-PAAm microgel could not be measured because of its submicron scale size, the elasticity of CD-PAAm microgel suggests to increase upon the addition of BPA. The rapid BPA-responsiveness of the CD-PAAm microgels compared to previously reported BPA-responsive bulk hydrogels was attributed to the large surface area of the CD-PAAm microgels. These BPA-responsive CD-PAAm microgels, prepared *via* inverse miniemulsion polymerization using a water-soluble PSB-POEG emulsifier, provide a useful platform for designing rapidly responsive soft nanomaterials for use in molecular sensors, separation substrates, and drug delivery carriers. Detailed studies on the kinetics of BPA-responsive shrinkage and changes in the mechanical properties of CD-PAAm microgels are currently under investigation.

## Conclusion

This paper reports the preparation of BPA-responsive microgels composed of a hydrophilic PAAm network with CD ligands for BPA *via* inverse miniemulsion polymerization using a water-soluble PSB-POEG emulsifier. The PSB-POEG emulsifier was synthesized *via* RAFT polymerization. The resulting PSB-POEG stabilized the water-chloroform interface in the W/O emulsion by decreasing the interfacial tension, similar to PMPC-*b*-POEGMA previously reported by our group. These results demonstrate the versatility of zwitterionic blocks in designing water-soluble emulsifiers. Because the PSB block exhibits the upper critical solution temperature (UCST)-type phase transition, the water-soluble PSB-POEG emulsifier will provide the platform to prepare UCST-type thermoresponsive soft nanomaterials. Water droplets in the W/O emulsions stabilized with PSB-POEG allowed the preparation of BPA-responsive microgels *via* inverse miniemulsion copolymerization of AAm, acryloyl-CD, and MBAA. The resulting CD-PAAm microgels exhibited rapid shrinkage in response to BPA owing to the formation of CD-BPA-CD complexes acting as dynamic cross-links, without nonspecific adsorption of BPA onto the hydrophilic microgel network. The proposed method for preparing hydrophilic microgels by inverse miniemulsion polymerization using a water-soluble emulsifier can be applied to prepare molecularly imprinted and bioconjugated microgels. In addition, the formation of molecular complexes between CD ligands and drugs, such as dapsone, doxorubicin, and curcumin, suggests that hydrophilic microgels with CD ligands can serve as platforms for designing drug carriers. Our proposed method for preparing microgels *via* inverse miniemulsion polymerization using a water-soluble emulsifier will contribute significantly to the development of molecular sensors, separation substrates, and drug delivery carriers.

## Supplementary Material

Supplemental Material
