# Supplementary material for: Bisphenol A-responsive microgel comprising hydrophilic poly(acrylamide) network
Source: Sci Technol Adv Mater. 2026 Jan 12;27(1):2610881. doi: 10.1080/14686996.2025.2610881 (PMC12854222; doi:10.1080/14686996.2025.2610881)
Supplement: Supplemental Material [file TSTA_A_2610881_SM8745.docx]

Supporting Information

Bisphenol A-responsive microgel comprising hydrophilic poly(acrylamide) network

Akifumi Kawamura^a, b^*, Fumiya Tanaka^a^, Yuriko Nishimura^a^, and Takashi Miyata^a, b^

^a^Department of Chemistry and Materials Engineering, Kansai University, Suita, Osaka, Japan

^b^Organization for Research and Development of Innovative Science and Technology, Kansai University, Suita, Osaka, Japan

*Akifumi Kawamura

E-mail: akifumi@kansai-u.ac.jp


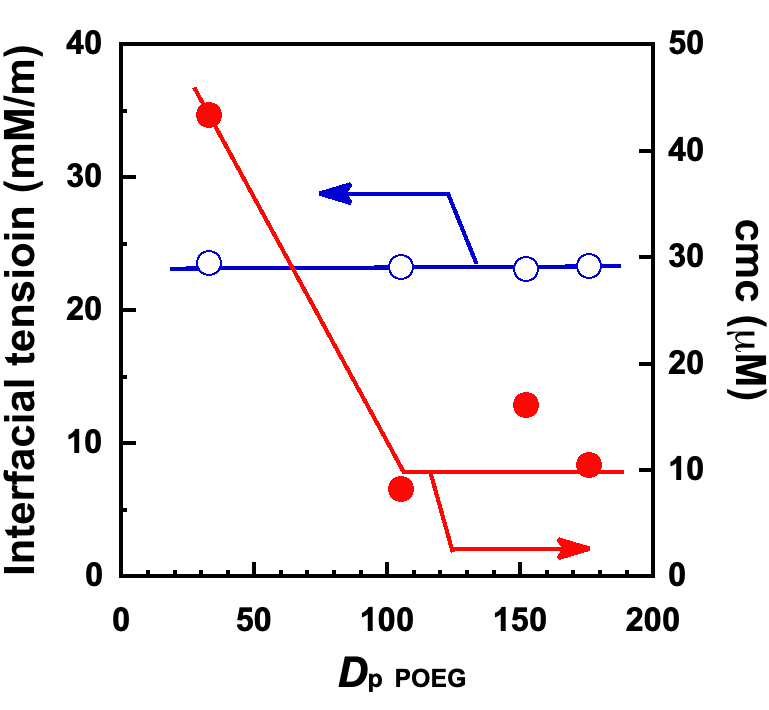


**Figure S1.** Effect of *D*_p_ _POEG_ on the interfacial tension (○) and CMC (●) of the water-chloroform two-phase system.


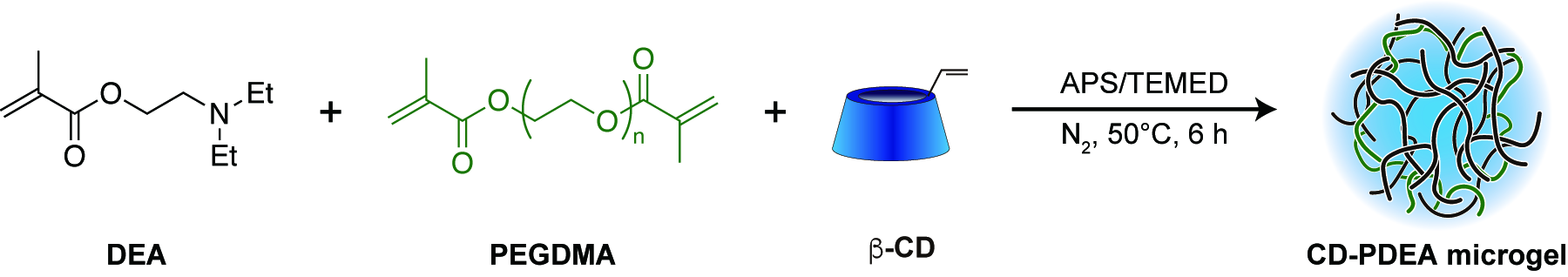


**Figure S2.** Synthesis of CD-PDEA microgels *via* soap-free emulsion polymerization.


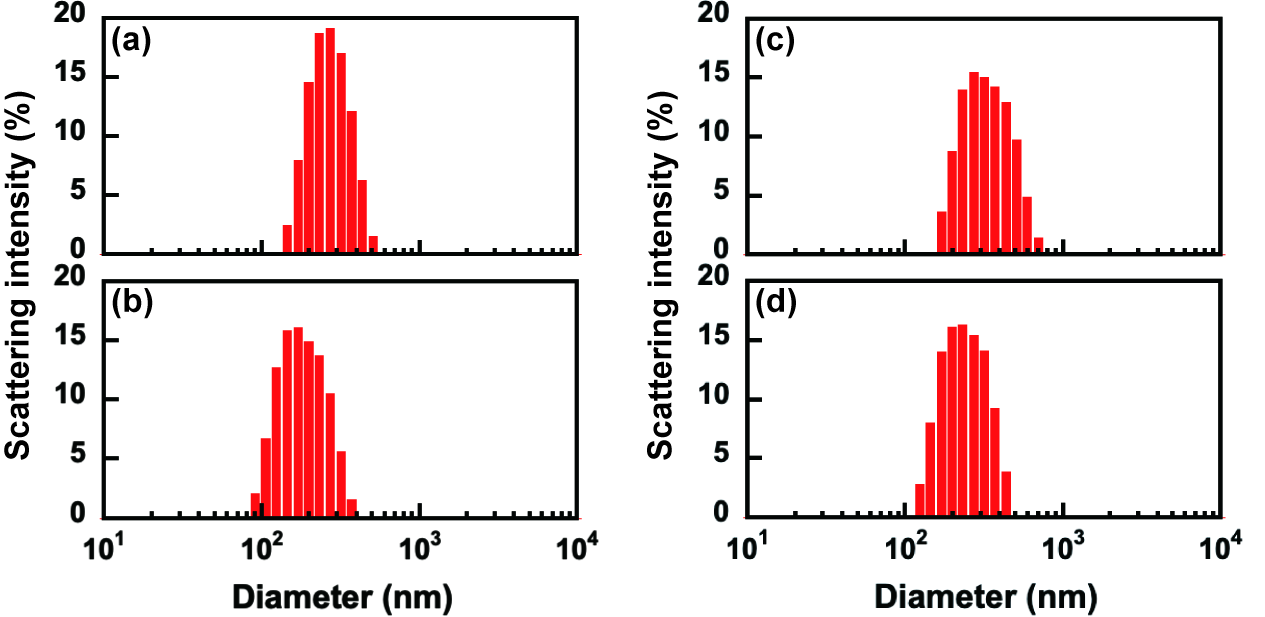


**Figure S3.** Size distributions of PDEA microgels before (a) and after the addition of BPA (c). Size distributions of CD-PDEA microgels before (c) and after the addition of BPA (d). The BPA concentration was 20 mg/L.

# Synthetic Method

## Synthesis of 4-[(3-methacryloyloxypropyl)dimethylammonio]butane-1-sulfonate (SB) [1]

An amount of 10.0 mL of acetone solution containing 1,3-propanesultone (7.77 g, 0.0636 mol) was added dropwise to 15.8 mL of acetone solution containing *N,N*-dimethylaminoethyl methacrylate (5.00 g, 0.0318 mol) for 30 min and then the mixture was stirred at room temperature for 24 h. The produced white solid was collected by filtration and washed with acetone (31.6 mL). The product was then dried under vacuum to obtain SB as a white solid (8.21 g, 92% yield)

^1^H NMR (400MHz, D_2_O) *δ* ppm: 6.17 (*s*, 1H, C*H*_2_=C–), 5.79 (*s*, 1H, C*H*_2_=C–), 4.66 (*m*, 2H, -C(=O)O-C*H*_2_–), 3.84 (*t*, *J* = 8 Hz, 2H, –C(=O)O-CH_2_-C*H*_2_-N^+^–), 3.60 (*m*, 2H, –N^+^-C*H*_2_-CH_2_SO_3_^–^), 3.23 (*s*, 6H, C*H*_3_-N^+^–), 2.99 (*t*, *J* = 8 Hz, 2H, –C*H*_2_-SO_3_^–^), 2.28 (*quint*, *J* = 8 Hz, 2H, –N^+^CH_2_-C*H*_2_-CH_2_SO_3_^–^), 1.95 (*s*, 3H, CH_2_=C-C*H*_3_)

## Synthesis of Acryloyl-modified β-cyclodextrin (acryloyl-CD)

Acryloyl-CD was synthesized via 5 step reaction by previously reported method as illustrated in Scheme S1 [2].

**Scheme S1.** Synthetic scheme of acryloyl-CD.

### Synthesis of p-toluenesulfonic anhydride (Ts_2_O)

*p*-Toluenesulfonyl chloride (40.13 g, 21 mmol) and *p*-toluenesulfonic acid monohydrate (20.05 g, 11 mmol) were dispersed in 250 mL of dichloromethane. The reaction mixture was stirred for overnight, and then the unreacted *p*-toluenesulfonyl chloride was removed by filtration. The filtrate was evaporated, and then the residue was recrystallized from isopropyl ether to yield Ts_2_O as a white solid; yield, 21.9 g (61 %).

### Synthesis of 6-O-monotosyl-6-deoxy-β-cyclodextrin (TsO-CD)

β-CD (27.66 g, 24.4 mmol) and Ts_2_O (11.64 g, 35.7 mmol) were dispersed in 200 mL of deionized water, and then the suspension was stirred for 2 h. 120 mL of NaOH solution (2.5 M) was added to the reaction mixture, and after 10 min the unreacted Ts_2_O was removed by filtration. The filtrate was neutralized by the addition of HCl, affording TsO-CD was collected after cooling at 4 °C for overnight; yield, 9.38 g (29 %).

^1^H NMR (400 MHz, DMSO-*d*_6_) δ ppm: 7.75 (d, *J* = 8.0 Hz, 2H, Ar–H), 7.44 (d, *J* = 8.0 Hz, 2H, Ar–H), 5.86–5.66 (m, 13H, O–H of CD), 4.84–4.19 (m, 14H, C–H of CD), 4.59–4.19 (m, 7H, O6–H of CD), 3.36–3.48 (m, overlaps with HOD), 2.09 (s, 3H, CH_3_–Ar).

### Synthesis of 6-deoxy-6-azide-β-cyclodextrin (CD-N_3_)

TsO-CD (3.80 g, 2.95 mmol) and NaN_3_ (2.49 g, 38.3 mmol) were dissolved in 555 mL of deionized water, and then stirred for 5 h at 80 °C, followed by pouring into acetone to precipitate the CD-N_3_ as a white powder. The obtained CD-N_3_ was dried in vacuo; yield, 2.63 g (77 %).

^1^H NMR (400 MHz, DMSO-*d*_6_) δ ppm: 5.74 (br, 14H, O–H), 4.88–4.54 (m, 14H, C–H of CD), 3.85–3.29 (m, overlaps with HOD).

### Synthesis of 6-deoxy-6-amino-β-cyclodextrin (CD-NH_2_)

CD-N_3_ (2.50 g, 2.16 mmol), triphenylphosphine (0.65 g, 2.48 mmol) were dissolved in 4.3 mL of DMF, and then the reaction mixture was stirred for overnight at room temperature. After the addition of 5.43 mL of water and 5 mL of DMF, the reaction mixture was stirred at 90°C for 2 h, and then reaction mixture was poured into acetone to precipitate CD-NH_2_ as a white powder. The obtained CD-NH_2_ was dried in vacuo; yield, 2.03 g (83 %).

^1^H NMR (400 MHz, D_2_O) δ ppm: 5.04 (s, 7H, C1–H of CD), 3.93–3.55 (m, 42H, C2, 3, 4, 5, 6–H of CD).

### Synthesis of acryloyl-6-amino-6-deoxy-β-cyclodextrin (acryloyl-CD)

CD-NH_2_ (1.01 g, 0.89 mmol) was dissolved in 15 mL of NaHCO_3_ aq. (pH 11). Acryloyl chloride (3.6 mL, 44.2 mmol) was added to the solution of CD-NH_2_ on ice bath. The solution was stirred for 3 h, and then the reaction mixture was poured into acetone. The precipitate was dried in vacuo to yield acryloyl-CD as a white powder; yield, 0.74 g (70 %).

^1^H NMR (400 MHz, DMSO-*d*_6_) δ ppm: 6.32–6.14 (m, 2H, CH_2_=CH–), 5.80–5.75 (m, 1H, CH_2_=CH–), 5.05 (s, 7H, C1–H of CD), 3.94–3.29 (m, 42H, C2, 3, 4, 5, 6–H of CD).

## Synthesis of PDEA and CD-PDEA microgel via soap-free emulsion polymerization

DEA, acryloyl-CD, and PEGDMA (*M*_n_: 330) were added to 5 mL of water and dispersed by sonication. Next, 0.1 mL of 3.2 M aqueous TEMED solution and 0.1 mL of 0.05 M aqueous APS solution were added to 5 mL of monomer dispersion, and then, the mixture was stirred for 6 h at 50 °C at 500 rpm under an argon atmosphere. The reaction mixture was poured into seamless cellulose tubing (molecular weight cut off = 12,000-14,000) and dialyzed against water to purify the resulting microgels. The synthesis conditions are summarized in Table S1.

**Table S1**. Synthesis condition of PDEA and CD-PDEA microgels

|  | DEA | acryloyl-CD | PEGDMA |
| --- | --- | --- | --- |
|  |  |  |  |
| PDEA microgel | 12 mg  (65 μmol) | – | 5.5 mg  (17 μmol) |
| CD-PDEA microgel | 12 mg  (65 μmol) | 4.03 mg  (3.4 μmol) | 5.5 mg  (17 μmol) |

# References

[1] Duann Y-F, Chen Y-C, Shen J-T, et al. Thermal induced graft polymerization using peroxide onto polypropylene fiber. Polymer. 2004;45(20):6839-6843. doi: 10.1016/j.polymer.2004.08.008

[2] Kawamura A, Kiguchi T, Nishihata T, et al. Target molecule-responsive hydrogels designed via molecular imprinting using bisphenol A as a template. Chem Commun. 2014;50(76):11101-11103. doi: 10.1039/c4cc01187b
